# Fruit Seeds with Functional Applications: From Food Waste to Potential Uses

**DOI:** 10.3390/molecules31101626

**Published:** 2026-05-12

**Authors:** Dayane Stéphanie Fernandes, Geovana Miyashiro Ferreira Neto, Giullia Gabrielli Galiciani, Rosângela dos Santos Ferreira, Lidiani Figueiredo Santana, Priscila Aiko Hiane, Valter Aragão do Nascimento, Arnildo Pott, Rita de Cássia Avellaneda Guimarães, Karine de Cássia Freitas

**Affiliations:** 1 Graduate Program in Health and Development in the Central-West Region of Brazil, Federal University of Mato Grosso do Sul, Campo Grande 79079-900, Brazil; dayane.fernandes@ufms.br (D.S.F.); giullia.g@ufms.br (G.G.G.); rosangela.ferreira@ufms.br (R.d.S.F.); priscila.hiane@ufms.br (P.A.H.); valter.aragao@ufms.br (V.A.d.N.); rita.guimaraes@ufms.br (R.d.C.A.G.); 2 Pharmaceutical Science, Food and Nutrition Faculty, Federal University of Mato Grosso do Sul, Campo Grande 79070-900, Brazil; geovana.miyashiro@ufms.br; 3 Medical School, State University of Mato Grosso do Sul (UEMS), Campo Grande 79115-898, Brazil; lidiani.santana@uems.br; 4 Laboratory of Botany, Institute of Biosciences, Federal University of Mato Grosso do Sul, Campo Grande 79070-900, Brazil; arnildo.pott@ufms.br

**Keywords:** food waste, dietary phytochemicals, dietary supplements, functional properties, healthy food, plant extracts, sustainable food system, waste management

## Abstract

Significant amounts of food waste come from fruit processing, causing economic and environmental impacts. The waste generated is a valuable source of compounds due to its concentration of nutrients, such as dietary fiber, vitamins, minerals, lipids with mono- and polyunsaturated fatty acids, and bioactive compounds. Despite the nutritional and functional qualities of the waste, it is still commonly discarded and underutilized, demonstrating the importance of studying it. The selected fruits described in this study are widely consumed by various populations around the world and are used at an industrial scale. The objective of this review is to discuss the use of seeds from grapes, passion fruit, melon, watermelon, papaya, guava, raspberry, and pomegranate and their benefits for human consumption. The seeds stand out for the possibility of oil extraction, creating a sustainable and healthy mode of production. Due to their nutritional composition rich in polyunsaturated fatty acids, they have been shown to be beneficial to health, promoting development, strengthening the immune system, and promoting the growth and maintenance of cell membranes, cardiovascular benefits, and antimicrobial and antioxidant activity, in addition to innovation in the cosmetics sector and applicability as biofuel. Therefore, the exploitation of this type of by-product shows promise in the search for alternative sources of vegetable oils and bioactive compounds with high added nutritional value and potential nutraceutical application, helping to increase the value of food waste and thus contributing to a better use of plant resources.

## 1. Introduction

Fruit processing, both on an industrial and domestic scale, results in the generation of solid waste which, when disposed of improperly, can cause considerable economic and environmental impacts [1]. According to the 2024 Food Waste Index report by the United Nations Environment Programme (UNEP), the equivalent of 1.05 billion tons of food waste was generated, with households accounting for 60% of food wasted in 2022 [2]. The production of fruits and vegetables generates up to 25–30% of waste/by-products [3].

Although often undervalued, this waste has significant nutritional value, containing vitamins, minerals, bioactive substances, and fatty acids in relevant concentrations [4]. Among these by-products, seeds stand out for their high nutritional value and potential for use in human food and other sectors [5].

In particular, fruit seeds contain high levels of lipids, making them viable sources of mono- and polyunsaturated fatty acids, which are essential for maintaining human health and are of growing interest to the food, cosmetics, and pharmaceutical industries [6]. The nutritional composition rich in polyunsaturated fats is highly promising for health, with the development and strengthening of the immune system, growth, and maintenance of cell membranes [7].

In this context, the use of food waste emerges as an effective strategy to reduce waste and reintegrate by-products into the production cycle, aligning with the principles of the circular economy [8]. The incorporation of “clean” or “green” technologies in food waste processing has proven to be a promising approach to promoting the sustainable use of natural resources and increasing the value of functional ingredients, resulting in low environmental impact [9].

Fruit components and their therapeutic applicability have gained ground because they are widely consumed and represent a significant part of the human diet, providing important amounts of fiber, vitamins, minerals, and phytochemical compounds (phenols, bioactive compounds, and carotenoids, among others) that perform various health functions, such as antioxidant, anti-inflammatory, anticarcinogenic, and hypolipidemic actions, among others [10].

Fruit seeds, which can account for 10% to 35% of the total weight of the fruit, are often discarded, despite containing lipids, insoluble fiber, phenolic compounds, sterols, and fat-soluble vitamins [11], with potential applications in various industrial segments. In the food industry, for example, seed oils have been incorporated into formulations such as breads, cakes [12], sauces, and yogurts, providing nutritional and functional benefits. Chaouch and Benvenuti [1] demonstrated that the addition of grape seed oil to bakery products resulted in a 22% increase in unsaturated fatty acid content, as well as improvements in product texture and stability. In addition, there is growing evidence that seed extracts benefit the intestinal microbiota, modulate lipid metabolism, and have antihypertensive activity, reinforcing their nutraceutical applicability [13].

In the cosmetics industry, seed oils such as watermelon (*Citrullus lanatus*) and pomegranate (*Punica granatum* L.) have been used for their emollient, antioxidant, and regenerative properties, offering oxidative stability and sensory acceptability [14]. These applications reflect not only the functional potential of these oils but also their ability to replace synthetic ingredients, contributing to more sustainable formulations.

From an environmental perspective, the use of seeds contributes to mitigating the impacts of agribusiness and urban garbage, promoting the rational use of natural resources and adding value to waste previously considered disposable. Malik et al. [15] show that the incorporation of plant by-products into innovative production chains, such as the manufacture of biofilms and biodegradable packaging, represents an effective strategy for replacing synthetic polymers with bio-based materials.

Interest in sustainable alternatives for agro-industrial waste management has driven research into plant matrices with high bioactive compound contents. Campos et al. [13] highlight that reintegrating waste into the production system can foster innovative value chains, contributing to the sustainable development of the agri-food sector. The incorporation of seed extracts into food formulations not only contributes to waste reduction but also allows the replacement of synthetic additives, thanks to their antioxidant and antimicrobial properties [16].

This practice promotes the reuse of waste as renewable sources of fiber, vitamins, and organic acids [15]. According to Sawarkar et al. [14], fruit seeds have physical and chemical characteristics that are favorable for the production of stable bioactive ingredients, being highlighted as sources of carbohydrates, lipids, and phenolic compounds, with potential application in food supplements and biodegradable materials.

The rational use of these biomasses is also related to food and nutritional security, since the extracted bioactive compounds act in the prevention of chronic diseases such as type 2 diabetes mellitus, obesity, and different types of cancer [17]. In addition, Patra et al. [18] demonstrated that mango (*Mangifera indica* L.), dates (*Phoenix dactylifera* L.), and orange (*Citrus sinensis* (L.) Osbeck) seeds have high concentrations of phenolic substances with significant antioxidant activity, making them promising as functional ingredients.

In this scenario, fruit seeds emerge as strategic elements in the construction of resilient food systems. The recovery of biomolecules from this waste enables the production of bioplastics, biofertilizers, and bioenergy, promoting an integrated approach to reuse [15]. Complementarily, Ansari et al. [19] demonstrated that the anaerobic digestion of agro-industrial waste, including seeds, can generate highly efficient bioenergy products, reaffirming the multifunctional potential of these raw materials.

Finally, the growing demand for functional ingredients with antioxidant and anti-inflammatory properties has stimulated the exploration of fruit seeds, traditionally considered by-products, as alternative sources of vegetable oils with high nutritional value and nutraceutical applications [1,13,18,20]. Advances in extraction technologies and recognition of the functional potential of these compounds consolidate seeds as strategic resources for innovation and sustainability in agribusiness. In view of this, this review aims to investigate the potential use of products obtained from the extraction of fruit seeds, emphasizing their contributions to health and the mitigation of the impacts of agribusiness and urban garbage, promoting the rational use of natural resources and adding value to waste previously considered disposable. The fruits selected for description in this study are widely consumed by various populations around the world, are used at an industrial scale, and, as a result, generate large-scale seed production, from which oils and bioactive compounds can be extracted for various applications in the cosmetic, pharmaceutical, and food industries.

This narrative, integrative review (covering both experimental and non-experimental studies) compiles various articles that examine the potential use of the fruit seeds identified as the focus of this study as health promoters, based on their nutritional components, as well as approaches related to biological activities and the sustainable use of food waste. The information for this review was collected from various scientific databases, such as PubMed, SciELO, Scopus, ScienceDirect, and Google Scholar. The following keywords were used: Food waste; Healthy food; Plant extracts; Dietary phytochemicals; Functional properties; Sustainable food system; Dietary supplements; and Waste management. They were used to assist in the search for articles, using selection criteria based on critical analysis of the texts and synthesis of the results.

## 2. Food Waste and Use of Food Residues

The Food and Agriculture Organization of the United Nations (FAO) defines food loss as unintentional loss, such as contamination caused by a specific situation. Food waste, on the other hand, is characterized as the disposal of food that is fit for consumption [21].

Food waste represents a comprehensive and underestimated challenge, with significant implications in several areas. Even though one can theorize about its impact on global food security, its full socioeconomic and environmental ramifications are evident. The interactions between food waste, natural resources, and social justice point to the need for substantial changes in food production and waste management, with a view to reducing inefficiencies and externalities. Given its complexity, food waste likely requires a combined approach of technological solutions and direct public sector interventions, along with incentive structures designed to modify food disposal behaviors [22]. Considering these conditions, the involvement of governments, businesses, and civil society is crucial, joining efforts to support the UN Sustainable Development Goals, established as global targets for 2030. Specifically, Goal 12 establishes ensuring responsible consumption and production through the adoption of sustainable practices, substantially reducing waste generation through prevention, reduction, recycling, and reuse throughout production and supply chains. This includes supporting developing countries by strengthening their scientific and technological capacities to achieve more sustainable production and consumption patterns, as well as the efficient use of natural resources [23].

**Figure 1:**
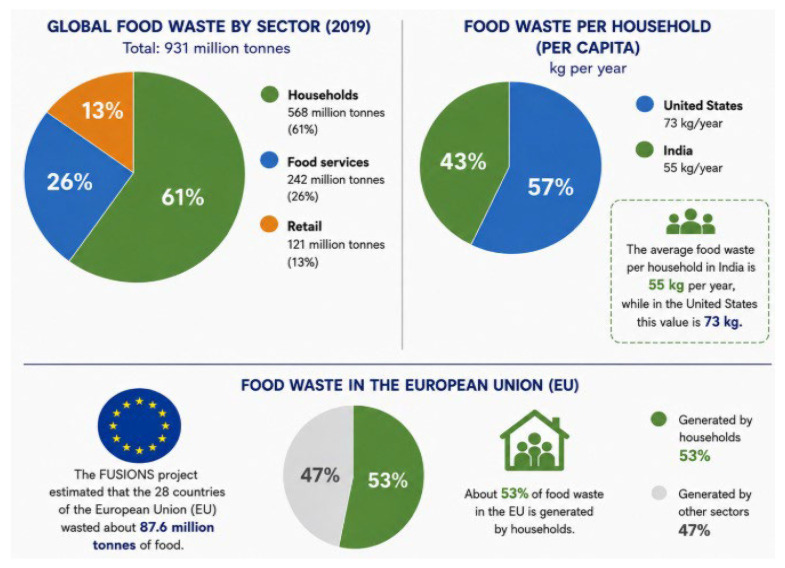
Representation of food waste, which occurs both on a global scale and at the household level. Source: Food Waste Index Report [24]; FUSIONS project [25].

The 2021 Food Waste Index Report [24] estimates that around 931 million tons of food waste was generated in 2019, 61% of which came from households, 26% from food service, and 13% from retail. According to the findings published in the United Nations Environment Programme’s Food Waste Report 2024 [2], the highest absolute figures for food waste were recorded in the two countries with populations of over a billion people, when looking at the data on a country-by-country basis. The scenario is rather different regarding waste produced per capita. For example, the average household in India discards 55 kg of food per year, while for the United States the figure is 73 kg. Figure 1 is an adaptation of the results presented in the UNEP Food Waste Index Report, 2024.

It is important to highlight the source of waste generated by the food industry. In this context, the fruit juice processing industry stands out, as it produces large volumes of organic waste, such as peels, seeds, and fibers, which also causes air, water and soil pollution through the decomposition of these materials [26]. In the wine industry, waste generation, when properly utilized, offers potential economic benefits in the production and preservation of food, nutraceuticals, and cosmetics, as well as environmental protection [27]. With a view toward resource utilization and sustainability, the processing of this food waste can be effectively harnessed to generate products for aerobic composting and fertilizers, and, in particular, for the production of bioenergy and biofuels, thereby promoting the bioeconomy [19]. The development of more environmentally friendly and sustainable solutions for food waste from the agro-industrial sector is a matter of concern and presents challenges for environmentalists and governments worldwide [26].

Such waste is related to a lack of information about the nutritional quality of food waste and parts commonly discarded such as leaves, peels, stalks, and seeds, as well as little guidance and knowledge about different ways of using and preserving them, ultimately discouraging the full consumption of food [28,29]. The use of food waste, such as peels, seeds, and stalks, not only minimizes environmental pollution and economic costs but also brings benefits to human health and helps prevent diseases [30], which is directly related to their nutritional and phytochemical composition, with high concentrations of vitamins, minerals, bioactive compounds, phenolic compounds, and fatty acid composition [4], in addition to fibers.

Seeds, although often discarded in the food industry only focused on juices, jams, and salads, have added nutritional value, combining physical and chemical characteristics favorable to the production of stable bioactive ingredients. They are highlighted as sources of carbohydrates, lipids, and phenolic compounds, with potential application in food supplements and biodegradable materials [4]. Thus, the adoption of strategies that take advantage of these seeds, not only as a source of nutrition but also as a sustainable measure, can represent effective alternatives while contributing to reducing the environmental impact caused by their disposal as waste [5].

Therefore, it is important to consider that food loss and waste are significant drivers of environmental pressure, compromising the transition to a sustainable food system. Food waste originating from stages involved in the food chain, such as agricultural production, food processing, distribution to points of sale, collective food services, and households, contributes significantly to environmental impacts. Originating from this supply chain, emissions of gases such as volatile organic compounds, ammonia, carbon dioxide, methane, and hydrogen sulfide are expected, which are harmful to respiratory and skin health, as well as contaminating the land and fresh and salt water, in addition to the eutrophication process [31,32].

Fruits and vegetables are major food categories that require significant amounts of agricultural inputs [33]. A study corroborated this statement, finding a relationship between a higher quality diet, consisting of more vegetables and fruits, and greater food waste, considering the lower land requirements and higher rates of use of nitrogen fertilizers and irrigation water [34]. However, with a view toward reducing waste throughout the food supply chain, it is essential to adopt waste management strategies to mitigate overall impacts, such as the depletion of groundwater and degradation of water quality [35,36]. This is in addition to employing technologies for gas capture, bioenergy and biofuel generation, thus diverting the harmful effects of food waste to benefits for human and environmental health [32].

## 3. Chemical Characterization and Quality of Vegetable Oils

Fruits such as grapes, passion fruit, melon, watermelon, papaya, guava, raspberry and pomegranate are widely used in the food industry and are also potential sources of waste. But at the same time, they have great potential for full utilization since. For example, their seeds contain a large proportion of lipids, which allows the extraction of their oils, and they are excellent sources of unsaturated fats, mainly monounsaturated (Table 1). In addition to the lipid fraction, these seeds contain bioactive compounds with high antioxidant capacity (Table 2).

There are a variety of methods in the literature for assessing the quality of oils and fats, checking for adulteration, improper handling, and characteristics that may interfere with the effects of the product due to loss of nutritional characteristics. Such quality assessment methods are measures adopted by organizations from different segments around the world to define standards in procedures, policies, and actions [49]. Table 3 presents some indices that reflect the results of quality assessments of oils extracted from the seeds of the following fruits: grape, passion fruit, melon, watermelon, papaya, guava, raspberry, and pomegranate.

For large-scale oil extraction from over 5 million existing plant species, only 12 are used, resulting in scarcity and a gap in the vegetable oil market. By using oil from parts of the plant that would otherwise be discarded, such as seeds, a sustainable and healthy production method is created [56,57], since agricultural waste is a source of great potential for raw materials for the generation of biofuels [58], natural sources of ingredients for the development of functional food products [57], and for the pharmaceutical and cosmetic industries [59].

The literature contains examples of oils that have been extensively explored by scientific studies. Among them is olive (*Olea europaea* L.) oil, renowned for its numerous health benefits, correlated both with its fatty acid composition and constituents like tocopherols, phenolic compounds, various minerals, and B vitamins, making it a rich source of natural antioxidants with high biological power [60]. Another example is soybean oil (*Glycine max* (L.) Merrill), used as a food source and, with new technologies, also as a biofuel. It is an excellent source of essential fatty acids, containing 50% linoleic acid, 7% linolenic acid, and 23.3% oleic acid [61].

There are also other commercially available oils, such as canola, safflower, corn, palm, sunflower, and coconut, which are notable for their nutritional qualities and potential health benefits. It should be noted that the quality of an oil is directly associated with its chemical composition, which depends on agronomic factors, climatic conditions, the variety and state of ripeness, technological factors related to extraction, such as the method and type of equipment used, as well as the conditions of harvesting, storage, and transportation of the product [62].

Improving knowledge about the composition of these fruit seeds and their oils makes it possible to further disseminate their use, contributing to sustainability, since products made from raw materials considered waste will be used.

## 4. Fruit Seed Oils

The oil content in seeds and the type of fatty acid vary considerably between species. In mango seeds, lipids represent between 10% and 15% of dry matter, while in grape seeds this value can reach up to 20% [1,18]. According to Babbar, Oberoi, and Sandhu [18], mango, dates, and passion fruit seeds have a lipid composition rich in essential fatty acids, such as oleic (29.6–45.3%) and linoleic acid (21.2–42.8%), in addition to relevant concentrations of tocopherols (up to 42 mg/100 g) and phytosterols, which confer cardioprotective properties. Complementarily, Avila-Román, Talero, and Motilva [20] identified that olive seed oil contains about 72.4% oleic acid, 10.8% linoleic acid, and 8.6 mg/100 g of α-tocopherol, as well as phenolic compounds such as tyrosol and hydroxytyrosol, associated with potent antioxidant activity (IC_50_ = 0.69 mg/mL).

The fatty acid profile of seed oils, in addition to being highly variable between species, is influenced by environmental factors such as climate, soil type, and fruit ripeness. Poureshaghi et al. [63], when evaluating ten Iranian grape cultivars (*Vitis vinifera* L.), observed a variation of 4.03% to 18.01% in oil content, with linoleic acid being the main fatty acid identified, varying from 17.9% to 59.4%. The seeds were rich in polyunsaturated fatty acids, which accounted for between 17.9% and 62.1% of total fatty acids, while monounsaturated fatty acids accounted for 3.7% to 16.8% and saturated fatty acids for 20.9% to 78.4%.

Chaouch and Benvenuti [1] reported that grape seeds contain about 70% linoleic acid, in addition to tocopherols and flavonoids, which promote the oxidative stability of the oil and contribute to the modulation of lipid metabolism. Malik et al. [15] emphasize that phytosterols present in oils, such as β-sitosterol and campesterol (0.8–1.6 g/100 g), reduce intestinal cholesterol absorption, justifying their use in nutraceutical formulations. In addition, the diverse phytochemical composition of these oils allows them to be used in the cosmetics industry, due to their moisturizing and regenerating properties [19].

For example, pomegranate seeds have been reported to contain concentrations of up to 150 mg GAE/100 g of extract, containing anthocyanins, flavonoids, and phenolic acids that inhibit lipid peroxidation and scavenge free radicals [13]. The antioxidant efficacy of these compounds is proven by IC_50_ values below 0.9 mg/mL in DPPH and ABTS assays, suggesting their applicability in cosmetic formulations and functional foods [17].

The efficient extraction of seed oils depends on the method used [64]. The most common traditional oil extraction methods used on an industrial scale are solvent extraction and extrusion pressing [65]. The use of organic solvents raises environmental concerns, but extrusion pressing yields less oil compared with the use of solvents [66].

Unconventional techniques such as supercritical fluid extraction, ultrasound, and microwaves have shown higher yields and lower thermal degradation of bioactive constituents compared with traditional methods [64]. The use of cellulolytic and pectinolytic enzymes has also proven effective in releasing lipids bound to the plant matrix, promoting cleaner extractions with a higher content of functional compounds [15]. In addition, oil encapsulation can be an efficient strategy to ensure oxidative stability and expand the industrial application of these ingredients, especially in products with extended shelf life [13].

The replacement of conventional oils with seed oils in food formulations has established itself as an alternative for the development of functional products. Sawarkar et al. [14] highlight that pumpkin seed oil (*Curcubita pepo* L.), watermelon seed oil, and pomegranate seed oil have lipid profiles that are beneficial to cardiovascular health, with high levels of polyunsaturated fatty acids. In addition, Sandhu et al. [16] demonstrated the antimicrobial and antioxidant activity of grape seed oil, suggesting its application as a natural preservative.

In olive seeds, for example, phenolic compounds such as elenolate and tyrosol have been identified, in addition to a rich and structurally complex lipid matrix [20]. Chaouch and Benvenuti [1] confirmed that grape seed oil contains catechins and linoleic acid in levels that can exceed 70%, reinforcing its functional potential.

**Figure 2:**
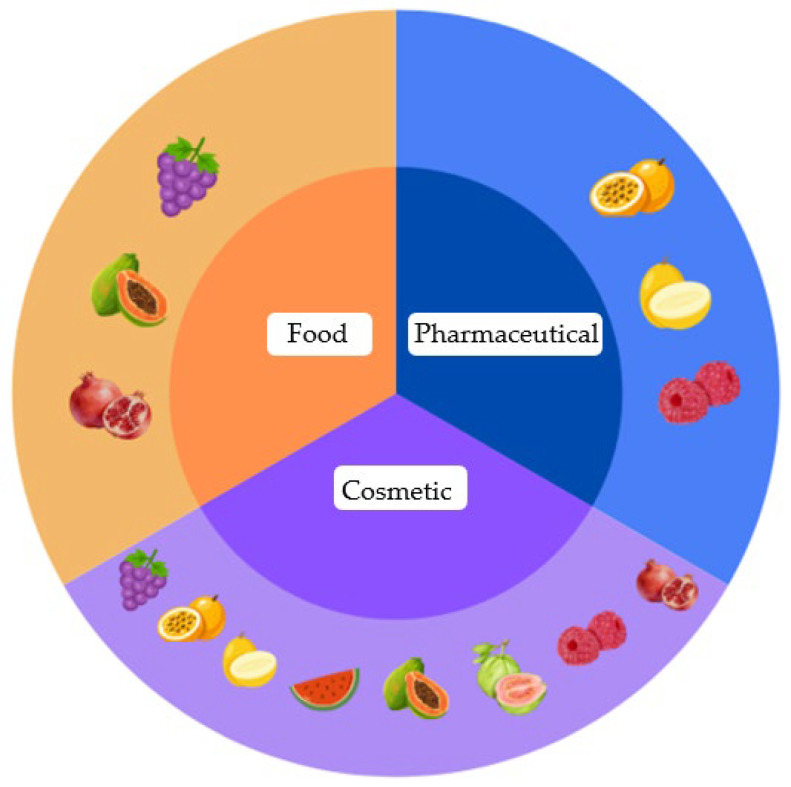
Categorization of fruit seed oils and their respective industries of use.

In this scenario, the use of seeds to obtain vegetable oils represents a strategic approach that integrates health, sustainability, and technological innovation. These oils act as vehicles for bioactive compounds and substrates for the development of new natural products with lower environmental impact and high functionality [17,19]. The use of these raw materials favors the advancement of the bioeconomy, with promising impacts on the production chain and the valorization of underutilized resources [13]. These products are commercially available in the form of oils for food, cosmetic and pharmaceutical use (Figure 2).

### 4.1. Oil and Compounds from Grape Seeds

*Vitis vinifera* can be called European grape, common grape, or grapevine. It originates from the Mediterranean region, covering Central Europe to northern Iran. Several subspecies are cultivated in temperate regions around the world. It is the most widely cultivated grapevine species for winemaking [67]. Grape seeds are a major waste product of the wine industry, given that current global wine production exceeds 290 million hectoliters per year, with Italy and France being the largest producers. Such huge production has a direct impact on the amount of waste produced and discarded [68].

The chemical composition of this residue is approximately 32% carbohydrates, 35% fiber, 12% protein, 7% to 20% lipids, 3% minerals, and 7% water. Cold-pressed oil extraction has linoleic acid, a polyunsaturated fatty acid (PUFA), as its predominant component, with oleic, palmitic, stearic, and α-linolenic acids also present [69]. Above all, grape extracts contain phenolic compounds, phytosterols [70], aromatic acids, flavonoids, proanthocyanidins and stilbenoids [71]. The biological activities of bioactive polyphenol molecules (resveratrol, quercetin, catechin/epicatechin, etc.) are found in large quantities in agricultural by-products, seeds, and peels of industrial relevance [72].

The effects of these bioactive compounds on human health have not been fully elucidated, but it is already known that polyphenols, plant antioxidants such as flavonoids, have positive effects on blood pressure, insulin resistance, lipid profile, systemic inflammation, cardiovascular and degenerative diseases, and tumors [73,74,75].

**Figure 3:**
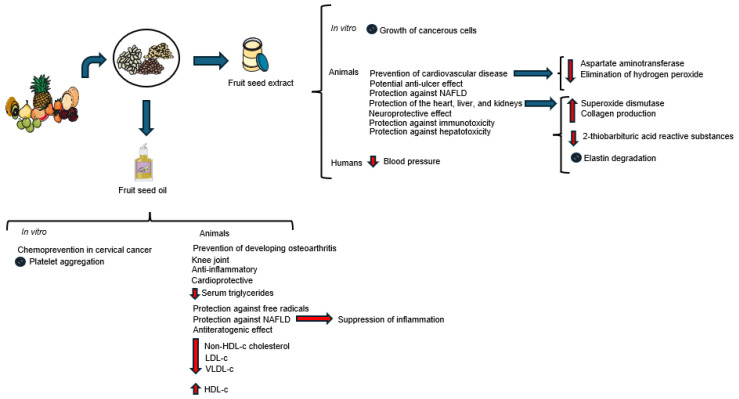
Effects of supplementation with fruit seed extract and oil on animal and human health (↓ decrese; ↑increase).

Several chronic diseases have their pathogenesis related to oxidative and inflammatory processes. In this sense, biological effects have been demonstrated in the use of grape seed oil and extracts (Figure 3).

*Vitis vinifera* seed oil showed anti-inflammatory and cardioprotective effects in an experimental study of isoproterenol (ISO)-induced ischemia in rats, administered at 4 mL/kg/day for 14 days. There was a significant reduction in ventricular conduction, the cardiotoxic effect of ISO on the ventricular myocardium, and in the levels of pro-inflammatory cytokines (interleukin-6, interleukin-1β, and tumor necrosis factor-α) and the cardiac enzyme creatine kinase. This cardioprotective effect is possibly due to the effect of catechins, procyanidins, and phenols. The highly notable bioactive action of phenolic compounds is due to the antioxidant and anti-inflammatory properties present in the oil of this seed [76].

Extracts made from grape seeds, leaves, or fruit, as well as grape juice, have antioxidant activities, and reports in the literature cite health benefits and prevent complications from stress and aging [77]. Grapes have a high polyphenol content, which are non-enzymatic antioxidants that act as a defense against free radicals and non-radical oxidants. Grape polyphenols have cardioprotective, anticancer, antidiabetic, anti-obesity, and anti-osteoarthritis effects on health and act through direct antioxidant properties or by modulating signal transducers [72].

Proanthocyanidins, phenolic compounds commonly found in grape seeds and derivatives, can modulate oxidative stress, nitric oxide levels, mitochondrial dysfunction, and apoptosis in conditions of hyperglycemia, showing antidiabetic activity [78]. Thus, studies have demonstrated the potential benefits of these compounds, with antidiabetic, anticarcinogenic, antiviral, vasoprotective, and neuroprotective effects of grape-derived flavonoids [55,72,79]. As in controlled trials with duration and dose–response analysis in adult humans, positive impacts arise with supplementation with grape seed extract, presenting hypotensive and heart-rate-reducing properties [80].

The use of grape seed oil via intra-articular injection (200 mg/day on days 1, 7, and 14 of the experiment), as well as oral administration (avocado and grape seed oil in a 2:1 ratio, 300 mg/day dissolved in 1–2 mL of distilled water for 10 weeks), had preventive effects on the development of knee osteoarthritis in an experimental rodent model [81].

Also, lipophilic grape seed proanthocyanidin (LGSP) showed anti-cervical cancer effects in vitro (HeLa cells) and in vivo (zebrafish xenograft model), inducing apoptosis and blocking cell cycles. There was inhibition of the antiproliferative effect in HeLa cells by an increase in the rate of apoptosis and the percentage of the G2/M phase in cells and a decrease in the growth of a HeLa xenograft tumor, showing a potential chemopreventive effect in cervical cancer [82].

Regarding toxicity assessment, a study with grape seed extracts, with different preparations and concentrations, showed no side effects, nor was there any reported increase in alanine aminotransferase (ALT) or hepatotoxicity [83]. Similarly, grape seed proanthocyanidin extracts, analyzed using the comet assay and micronucleus technique, were shown to reduce cytotoxicity and genotoxicity [84].

### 4.2. Passion Fruit Seed Oil and Compounds

Passion fruit is native to America and grows in tropical and subtropical regions. Only two varieties are commercially important: *Passiflora edulis* Sims f. *flavicarpa* Deg. (yellow passion fruit) and *P. edulis* f. *edulis* Sims (purple passion fruit) [85]. Brazil is the world’s largest producer and consumer of passion fruit juice. In 2023, approximately 700,000 tons of yellow passion fruit were produced [86]. One of the great potentials of this fruit is juice production, but this production generates a significant amount of waste, including seeds and peels.

Seeds represent 4 to 12% of passion fruit, consisting of about 30% oil [87]. However, most passion fruit seeds are discarded as unusable, and their nutritional and health values remain untapped. Therefore, passion fruit seeds as a raw material for the development of functional oil have great market potential and development prospects [88], and it is very important that solutions for their use be proposed.

The use of passion fruit seeds for the production of vegetable oil makes it possible to generate an economical and ecological product, and studies that extracted their oil found a good yield, ranging from approximately 18% to 33% lipids [89,90]. It should also be noted that the extraction method and the species of fruit can cause variation in oil yield. Thus, Ramaiya et al. [91] demonstrated that the lipid content varied from 24.34% to 29.65% in *P. edulis* (purple) using petroleum ether extraction. The oil yield in cold pressing, on the other hand, was 23 to 28% [92]. Regardless of the extraction method or species, this high percentage of oil makes passion fruit seeds have clear potential for the oil industry.

The oil extracted from these seeds has a high content of unsaturated fatty acids (87.54%) [93], demonstrating that this product has good potential for use in both human and animal nutrition, as well as in the cosmetics industry. This oil is composed mainly of linoleic acid, followed by oleic, palmitic, stearic, and arachidic acids. The seed therefore contains two essential fatty acids (linoleic and linolenic acid), with the linoleic acid content (73.14%) being higher than that of linolenic acid (0.41%) [87]. In smaller quantities, it is also possible to find palmitoleic, α-linolenic, myristic, heptadecanoic, lauric, and caprylic acids [52].

Fatty acid analysis by other authors also observed a predominance of linoleic fatty acid (72.69%), which is far higher than that of linolenic acid (0.26%) [94]. Piombo et al. [95] studied the seeds of *P. edulis* f. *flavicarpa*, finding 73.4% ω-6, linoleic acid, 209 ± 8.0 mg/100 g of total phytosterols, including campesterol, stigmasterol, β-sitosterol, and δ-5 avenasterol, and 465 ± 8.4 μg/g of total tocopherols, including α-tocopherol, β-tocopherol, γ-tocopherol, and δ-tocopherol, justifying the use of passion fruit oil in the food and cosmetics industries.

The use of oils from seed waste is well regarded as a sustainable alternative for energy production because it does not compete with food production and provides an environmentally friendly use for waste. Passion fruit seeds are produced on a large scale by the food industry. The extraction and characterization of oil from passion fruit seeds has demonstrated its applicability as a biofuel, since it is possible to extract oil from this waste.

The biodiesel obtained from this oil met the parameters specified for biofuel in Brazil, although it requires additional treatment to remove oxygenated compounds and thus become suitable for use as a diesel-like biofuel [96].

In the same line of study and considering that one of the main concerns in the use of biodiesel is its susceptibility to radical-mediated oxidative reactions, the use of methanolic extract from passion fruit seeds was effective as an antioxidant additive for biodiesel, proving the usefulness of phenolic-rich plant extracts as additives. The presence of unsaturated methyl esters makes biodiesel vulnerable to oxidation, making the use of antioxidant additives useful. Passion fruit seed extract contains a high proportion of phenolic compounds (74.9 mgGAE/g) and exhibits excellent antioxidant activity. At a concentration of 200 ppm, passion fruit seed extract increased the fuel’s resistance to oxidation to levels where it could meet European and Indian specifications for blended biodiesel. The performance of passion fruit seed extract as an antioxidant additive for biodiesel was comparable to that of synthetic antioxidants, including butylated hydroxyanisole and butylated hydroxytoluene [97].

The use of ethanolic extract from passion fruit seeds (500 mg/kg body weight), due to its polyphenol content, had a protective effect on the heart, liver, and kidneys of rats subjected to oxidative stress induced by streptozotocin for a period of 15 days, showing increased levels of superoxide dismutase and decreased levels of substances reactive to 2-thiobarbituric acid. It also showed an effect on increasing collagen production and inhibiting the degradation of elastin and collagen, which may contribute to maintaining the structure of the dermis [98].

Piceatanol is abundant in passion fruit seeds and has been shown to have the potential to suppress the expression of matrix metalloproteinase MMP-1 (an enzyme responsible for the degradation of collagen fibers) induced by UV in fibroblasts, as well as inhibiting the Janus kinase 1 (JAK 1) signaling pathway, possible components responsible for inhibiting photoaging of human skin [99].

Considering the findings related to the extraction of oil from passion fruit seeds, it can be concluded that its use as a raw material is possible in various industrial segments, including the food industry, cosmetics, vitamin supplements, and biodiesel, in addition to other potential uses, particularly if we consider that the amount of waste generated is quite bulky. The relevant amount of antioxidants can serve as a natural food source of antioxidants, helping to prevent diseases, or as a food additive, increasing the stability and quality of food products, prolonging the shelf life. Thus, it is possible to add value to products that have been most frequently discarded as waste by extracting oil from passion fruit seeds, reducing waste and protecting the environment [92,96,100].

### 4.3. Melon Seed Oil and Compounds

The annual production of melon (*Cucumis melo* L.) worldwide is equivalent to more than 30 million tons, with China being the largest producer. This fruit has edible seeds that are used in recipes in some countries, such as India and Arabia, and represent up to 10% of its total weight [39]. The seeds are rich in lipids and proteins, with about 28% of the former and 30% of the latter in their composition. Fiber ranks third, with approximately 25%, followed by carbohydrates, 24%, and ash, 5% [52,101]. These results are influenced by the melon cultivar studied [52].

The growing search for new vegetable oils and the excellent oil content of melon seeds represent a valuable opportunity for industrial production. However, the choice of extraction method is essential to maintain product quality. The use of mechanical presses for cold extraction has resulted in the production of high-quality, affordable oils [52].

Crude oil from melon seeds is mainly composed of polyunsaturated fatty acids, 71.3% [102], predominantly linoleic and oleic acids, as well as high concentrations of vitamin E, making it a rich source of tocopherols and tocotrienols [52]. It also contains palmitic and stearic acids and, to a lesser extent, arachidic, α-linolenic, gadoleic, palmitoleic, heptadecanoic, lignoceric, myristic, pentadecanoic, behenic, cis-10-heptadecanoic, and eicosadienoic acids [39].

The presence of these fatty acids highlights the nutritional and functional value of melon seeds. They may contribute to health when included in food formulations, helping with the dietary intake of essential fatty acids and promoting cardiovascular health [103]. Melon seed oil supplementation can reduce plasma cholesterol, promote fecal acid excretion and modulate the intestinal microbiota (Figure 3). Hao et al. [102] observed a 24% reduction in plasma cholesterol in hypercholesterolemic hamsters supplemented with 9.5% of this oil in their diet and a 150% increase in fecal bile acid excretion. This supplementation increased the gene expression of hepatic cholesterol 7α-hydroxylase (CYP7A1) and increased the production of short-chain fatty acids in feces, favoring the modulation of the intestinal microbiota.

The main cause of myocardial changes, whether structural or functional, is myocardial infarction. The use of extract obtained from melon seeds has been shown to protect myocardial tissue. Ethanolic extract from melon seeds promoted a dose-dependent reduction in pulse pressure, systolic pressure, diastolic pressure, mean arterial pressure (MAP), and heart rate in normotensive rats. When used at doses of 0.3 mg/kg, it reduced MAP to 65.7 ± 1.79 mmHg; when used at a dose of 0.5 mg/kg the reduction was 53.28 ± 1.20 mmHg; and when administered at 1.0 mg/kg to 44.4 ± 0.43 mmHg, it presented a hypotensive response [104].

When a hydroethanolic extract of melon seeds was used in rats with acute hypertension, there was protection from chronic myocardial damage induced by isoprenaline (ISO), reducing inflammation and fibrosis without significant damage to myocardial tissue, except at low doses (75 mg/kg), pointing to possible protection from induced myocardial infarction. At doses of 150 mg/kg, there was a reduction in biometric indicators (heart diameter, heart weight, heart weight index, LVH weight and thickness, tibia length index, tail length index, and LVH index) [104]. This extract showed potent prevention of ISO-induced myocardial infarction. The results demonstrated vasodilator response and hypotensive effects in normotensive rats. As well as reducing cardiomyocyte hypertrophy and reversing changes in gene expression, biochemistry, and metabolism in rats with ISO-induced myocardial infarction, it reversed oxidative stress, energy consumption, and amino acid metabolism induced by infarction. However, more detailed studies involving humans are needed to establish the safety and efficacy [104].

Antiteratogenic and cardioprotective properties (Figure 3) were observed with the use of a methanolic extract from melon seeds in male rats with obesity induced by a high-fat diet. The use of 50, 100, and 200 mg/kg of extract promoted a significant decrease in body weight, body mass index, brown fat, white fat, fat mass, adiposity index, relative fat mass weight, serum triglycerides, LDL-c, and total cholesterol and an increase in HDL-c. Therefore, administration of the extract improved the lipid profile in experimental studies [105].

Melon is considered a medicinal plant with many antitumor and antioxidant compounds and may act as an effective medicinal supplement in cancer treatment. Gege-Adebayo et al. [105] observed that melon seed powder extract can inhibit the expression of genes related to angiogenesis and increase necrosis in tumor tissue in BALB/c mice, proving to be a good source of natural active components.

The use of melon seeds, an agro-industrial residue, has the potential to aid human health due to their fatty acid composition, which acts to control cholesterol metabolism, which is associated with the occurrence of cardiovascular diseases [39]. The extraction of oil from melon seeds is feasible for obtaining high-quality oil [52], and the use of the extract, whether ethanolic or methanolic, has shown beneficial results for cardiovascular health [104] and lipid profiles [105].

### 4.4. Watermelon Seed Oil and Composition

Watermelon (*Citrullus lanatus*) is a fruit highly appreciated worldwide, and in some parts of the globe, its seeds are used to produce edible oil, such as in Namibia, West Africa and the Middle East. In Europe, the oil obtained from the seeds is mainly used in the cosmetics industry [106]. The seeds can be easily removed from the fruit [40] and are often discarded, despite their potential applications [107].

Their seeds are rich in lipids, which, when extracted by cold pressing, can reach more than 40%, making them a highly sustainable resource for commercial exploitation [40]. They are also rich in protein, containing 28% to 37% [40,57]. They also contain 14% fiber, 10% digestible carbohydrates, and 4.45% ash [107]. They can be considered high in antioxidants and phytochemicals, such as flavonoids, vitamins, and polyphenolic compounds [57].

Watermelon seed oil is rich in linoleic and oleic fatty acids, which are considered essential fatty acids, as they are not synthesized by the human body, and also contains arachidic, palmitic, and stearic acids [40]. The oil contains tocopherol, predominantly γ-tocopherol, with 59.99 mg/100 g of oil in cold-pressed extraction, known as the most effective antioxidant among tocopherols, giving the product potential natural antioxidant action capable of stabilizing free radicals [108].

The phenolic compound content averages 35.72 µg GA E/mL, the total antioxidant activity averages 130.20 µg BHT Eq/mL, and flavonoids vary from 29.7 µg QE Eq/mL to 136.1 µg QE Eq/mL depending on the watermelon genotype, indicating potential antioxidant activity [57].

The phytochemical composition also includes tannin (8.95 µg/g ± 0.09) with the highest concentration, followed by oxalate (6.75 µg/g ± 0.06) and phytate (1.45 µg/g ± 0.09) with the lowest concentration. These are considered antinutritional compounds, but at low or moderate levels they do not cause harm to health and may even help protect against cardiovascular disease risks, as is the case with phytate at moderate levels, as it has an affinity for zinc and reduces the zinc/copper ratio in plasma. In terms of vitamin composition, vitamin C was found to be present at a level of 372.90 ± 1.91 mg/kg, which may help the immune system [106].

The effects of the numerous nutritional, antioxidant, and phytochemical properties of seeds on health are still limited in the literature. The presence of their nutritional constituents shows that it is useful to include this food in food fortification [107].

The antiteratogenic potential (Figure 3) was observed when watermelon seed powder was used in male mice knockout for the low-density lipoprotein receptor (LDL-r-KO). The animals showed a 58% reduction in the average size of atherosclerotic lesions, an effect that was associated with increases in average plasma levels of cytokines such as interleukin 10 (IL-10) and EPO (erythropoietin). Thus, there is evidence of a strong antiteratogenic property that may be mediated by changes in inflammatory pathways [109].

The use of diets containing watermelon seeds at doses of 2.5% or 5% in male and female Wistar rats showed possible negative effects on organs such as the kidneys and testicles. Oyenihi et al. [110] sought to investigate potential toxicity through chronic use, as consumption is associated with health benefits, but further investigation of possible undesirable effects is still needed. They observed an increase in testicular weight, sperm morphology abnormalities, a reduction in ovarian weight, and elevated serum urea and creatinine levels [110]. The assessment of the potential for toxicity in chronic use needs to be investigated, and studies need to be extrapolated to human health.

### 4.5. Papaya Seed Oil

Papaya (*Carica papaya* L.) is a perennial herbaceous plant with milky latex that can reach up to 12 m in height, producing fruit throughout the year [111]. Each fruit weighs between 1000 g and 3000 g, producing about 500 g and 1500 g of seeds [112]. Among 300 varieties of papaya, in Brazil, the fruits of the cultivar Papaia (Sunrise Solo) weigh less than 1000 g, and those of the cultivar Formosa exceed 3500 g. The oil extracted from the seeds has a fatty acid profile with significant amounts of oleic acid (71.3%), palmitic acid (16.16%), linoleic acid (6.06%), and stearic acid [42,57,58,113]. Papaya seeds also contain high levels of antioxidants, such as vitamins A, C, and E, which can mitigate pro-oxidant substances through various signaling pathways that promote the expression of antioxidant enzymes or reduce the production of reactive oxygen species (ROS) [114]. They have been used as a vermifuge for centuries, and the crude extract has been shown to contain a bioactive substance called benzyl isothiocyanate, which is probably the source of the antihelminthic activity of these seeds [115,116]. The main constituent of the essential oil extracted from papaya seeds is benzyl isothiocyanate [117].

Other biological applications of papaya seeds have been reported, such as in an experimental study in rats, which investigated immunostimulatory effects in combating immunotoxicity, concluding the potential health benefits of incorporating papaya seeds into food products, promoting immune system health, and protecting against liver damage [118]. Furthermore, they are considered a new natural source of antifungal agents, as they have promising antifungal activity, given that they have shown an inhibitory effect against Candida strains.

Santana et al. [119] observed that papaya seed oil had hypocholesterolemic, hypotriglyceridemic, and hypoglycemic effects in mice fed a high-fat diet (Figure 3), resulting in reduced levels of total cholesterol, non-HDL cholesterol, LDL-c, and VLDL-c, with an increase in HDL-c. In addition to hypoglycemic effects with lower fasting blood glucose values, it also protected against insulin resistance. Regarding the nutritional quality index, papaya seed oil exhibited low atherogenicity and thrombogenicity indices and an adequate omega-6:omega-3 ratio, showing potential contribution to the control of atherosclerotic diseases.

A study using papaya seed flour in the preparation of cupcakes offered to rats with immunotoxicity demonstrated improvement in the antioxidant system, inflammatory markers, and immunological parameters, returning them to normal levels and showing a reduction in hepatocellular necrosis and hepatocellular regeneration [120]. The use of freeze-dried aqueous papaya seed extract in a preclinical trial with albino rats demonstrated a neuroprotective effect against brain aging or age-related brain deterioration, with a reduction in depression and anxiety and an increase in muscle mass [121]. These properties may be promising in the incorporation of papaya seed residues into dietary supplements, acting as an adjunct in the control of inflammatory diseases.

As for toxicity assessment, acute toxicity studies in rats confirm the safety of oral administration of ethanolic extract from papaya seeds over a 14-day observation period, with no adverse effects observed at a dosage of 2000 mg/kg body weight. However, more complex, longer studies in different biological matrices, including at varying doses, are recommended to establish its safety profile and unlock its full potential for health and nutrition [122].

Therefore, the use of papaya seeds can provide a food matrix base for products in the food industry, being an alternative that can add value, in addition to reducing the disposal of agro-industrial waste [123].

### 4.6. Guava Seed Oil and Compounds

Guava (*Psidium guajava* L.) is a tropical fruit widely cultivated in regions of Latin America and Asia, generating seeds that correspond to about 12–15% of the fruit’s mass, traditionally considered agro-industrial by-products but recognized as relevant sources of lipids, proteins, fibers, and substances with bioactive action of nutraceutical interest [124]. The full use of these seeds is in line with sustainability and circular economy guidelines, transforming waste into high-value-added ingredients for the food, pharmaceutical, and cosmetic industries [125].

The lipid composition of the oil extracted from guava seeds shows a predominance of unsaturated fatty acids, with linoleic acid ranging from 55 to 66% and oleic acid from 14 to 24%, while the saturated content, mainly palmitic acid, is around 8–12% [126]. The oil extracted from the seeds contains up to 15% essential fatty acids, predominantly linoleic acid, which is responsible for cardioprotective and lipid-metabolism-modulating effects. In addition, significant amounts of oleic acid, palmitic acid, and phytosterols give the oil a nutritional profile comparable to that of other high-value-added vegetable oils [127,128].

This profile is similar to grape seed oil, which contains about 70% linoleic acid, but superior to tropical oils such as babassu (*Attalea speciosa* Mart.) oil, in which saturated fats predominate above 80% [128]. The poly:monounsaturated ratio, close to 2.6:1, suggests benefits associated with cardiovascular health and technological applications in food [129]. The presence of tocopherols, at levels above 20 mg/100 g of oil, contributes to protection against rancidity and reinforces its antioxidant potential [130].

In the cosmetics industry, the combination of unsaturated fatty acids and natural antioxidants gives the oil emollient, photoprotective and anti-inflammatory properties, making it promising in cream and lotion formulations. In parallel, there is growing interest in the development of nutraceutical supplements from seed extracts, especially aimed at cardiovascular health and the control of systemic inflammation [131], and the oil extracted from guava seeds presents such a formulation.

In addition to the lipid fraction, the seeds contain 10–15% protein on a dry basis, with a predominance of essential amino acids such as leucine, valine, and lysine, which highlights the possibility of application in food supplements and functional protein products [132,133]. The fibrous fraction represents approximately 30% of the seed, being mostly insoluble, with potential for application as a prebiotic ingredient capable of modulating the intestinal microbiota and assisting in gastrointestinal transit [133,134].

The n-hexane extract of guava seed oil contains a relevant concentration of phytosterols, notably β-sitosterol (297.61 mg/100 g), campesterol (11.04 mg/100 g), and stigmasterol (0.22 mg/100 g). These compounds are associated with the ability to reduce cholesterol absorption, potentially contributing to hypolipidemic and cardioprotective effects. The presence of ethyl esters, such as ethyl palmitate and ethyl linoleate, complements the lipid profile of the oil, indicating functional and pharmacological potential [128].

The phenolic fraction of guava seed oil revealed the presence of different bioactive substances identified by HPLC-ESI/MS, including catechin, isoquercitin, eriodictiol and quercetin, in addition to derivatives of chlorogenic acid, caffeic acid and ellagic acid. The total phenolic content was 45.57 ± 0.97 µg GAE/g of oil, indicating a relevant antioxidant potential capable of acting in the neutralization of reactive oxygen species (ROS) and in cell protection against oxidative damage [72].

The use of polysaccharides from guava seeds shows potential for inhibiting the growth of PC-3 prostate cancer cells through in vitro immunotherapy, as they possess GSF 3 polysaccharides that have been shown to be capable of preventing the growth of cancer cells through their pro-apoptotic gene expressions and alterations in the secretion of Th2-polarized and anti-inflammatory interleukin-10 cytokines secreted by immune cells (Figure 3) [135]. When administered directly to growing PC-3 cells, GSF 3 polysaccharides showed a maximum inhibitory concentration (50%) in these cells during 48 h of incubation at 320 µg/mL, suggesting cell growth inhibition. When used indirectly in conditioned medium of immune cells, it increased the inhibitory effect on splenocytes, probably by modulating the secretion of Th1/Th2 cytokines from splenocytes. There was an increase in pro-apoptotic effects in the Bax/Bcl-2 apoptotic signaling pathway, with an increase in the proportions of mRNA expression of this pathway in the treated cells [135]. Therefore, the use of polysaccharides may have the potential to inhibit differential tumor cells by inducing apoptosis, whether isolated or combined.

Maceration of guava seeds in methanol and dilution to 0.5%, with oral administration of 300 mg/kg to a model of indomethacin-induced gastric ulcer in male albino rats, was associated with anti-ulcer capacity by significantly decreasing ulcerogenic effects and protecting the mucosa from lesions, thereby assisting in healing: this effect was attributed to sterols such as stigmasterol and campesterol [136].

Therefore, the use of guava seeds, a food waste product, can contribute to reducing the environmental impacts of the guava production chain while generating new inputs with added value, aligning with the Sustainable Development Goals (SDGs) related to food security and the circular bioeconomy [137].

### 4.7. Raspberry Seed Oil and Derivatives

The raspberry (*Rubus idaeus* L.) is a fruit widely consumed throughout the world. Its world production reached more than 680 thousand tons in the year 2020, with Russia, Poland and the United States being the largest producers, respectively [138]. A large part of the production destined for raspberry wines and juices results in residues, such as pomace and seeds, which are underexploited but with great potential for use [139].

The seeds of this fruit have a good lipid content, mostly polyunsaturated, and can contain up to 23% oil, approximately 12% protein, and a large amount of antioxidants [139,140]. Palmitic, stearic, oleic, linoleic (ω-6), α-linolenic (ω-3), and arachidic acids are present in this oil, with linoleic acid contributing 49.9% of the total fatty acids and α-linolenic acid 25.98% [141].

Raspberry seed oil has carotenoids in its chemical composition, ranging from 0.81 mg/100 g to 3.25 mg/100 g depending on the extraction method, with β-carotene accounting for 43.21% of the total amount of carotenoids [139]. It has a high concentration of total tocopherols (185.1 mg/100 g oil), rich in γ-tocopherol (134.62 mg/100 g oil) and α-tocopherol (42.73 mg/100 g oil). Its total phenolic content is 22.4 ± 0.25 mg GAE/100 mg of oil and flavonoids are 1.34 ± 0.15 mg QU/100 mg [141].

These characteristics spark interest in studies testing the effects of this seed and its derivatives. Nonalcoholic fatty liver disease (NAFLD), being a serious health problem affecting the liver, characterized by the accumulation of fat in hepatocytes without a history of excessive alcohol consumption and involving numerous inflammatory processes, is the subject of numerous studies offering seeds and their derivatives. In the case of treatment with raspberry seed oil, it improved lipid parameters, reduced insulin resistance and glucose levels, suppressed inflammation, and modulated leptin, PPARγ (a transcription factor expressed in adipose tissue and liver), and adiponectin levels in rats (Figure 3). This therapy showed the preventive and protective action of raspberry seed oil against NAFLD induced by a high-fat diet (HFD), effects which may be due to the high content of unsaturated fatty acids and tocopherols and the adequate amount of phenolic compounds [141].

Redox-sensitive transcription factors, such as NF-κB, one of the main regulators of inflammation by activating the release of inflammatory mediators, can be activated by prolonged oxidative stress [142]. Raspberry seed oil significantly decreased the level of NF-κB in an experimental study with rats, thus exerting beneficial effects in suppressing inflammation [141] and consequently contributing to insulin action, since the increase in inflammatory cytokines contributes to insulin resistance [143].

The use of the oil in NAFLD, administered orally to male Wistar rats three times a week for 8 weeks, demonstrated the ability to improve the lipid profile, with a significant reduction in total cholesterol, triglycerides and LDL-c levels and an increase in HDL-c. It also showed an effect on liver enzyme activity, with a reduction in ALT and AST, in addition to suppressing inflammation through the inhibition of NF-kB, concomitant with a reduction in leptin levels, that is, acting with a protective and preventive effect against NAFLD induced by a high-fat diet [141].

The use of dried and ground raspberry seeds added to the diet (7.0 g/100 g of diet) of normotensive male Wistar-Kyoto rats and spontaneously hypertensive rats for a period of 6 weeks resulted in a reduction in the hepatic enzyme aspartate aminotransferase (0.9 times) and in the ability to eliminate hydrogen peroxide (reduction of catalase by 0.9 times). The decrease in catalase activity may indicate a decrease in the elimination of hydrogen peroxide, since catalase converts harmful hydrogen peroxide into water and oxygen. In supplemented normotensive rats, there was a 0.8-fold decrease in the atherogenic index, while nitric oxide synthase and prostacyclin synthesis, derived from the non-selective cyclooxygenase inhibitor indomethacin, increased acetylcholine-induced vasodilation [144], which may be considered for use in mechanisms for the prevention of cardiovascular diseases.

### 4.8. Pomegranate Seed Oil

Pomegranate (*Punica granatum* L.) is widely cultivated in countries such as India. Up to 25% of its total production consists of its seeds, which are normally discarded as waste. Its seeds are composed of approximately 12% water, 7% protein, 2% ash and 25% fiber. They are rich in lipids, reaching up to 20% yield, which facilitates the extraction of its vegetable oil [145,146,147,148]. Most of its lipid composition is punicic acid, which can vary between 48.91 and 82.81%. It is a PUFA, which makes it a unique edible oil in nutritional terms, with health benefits [149].

Punic acid, an omega-5 isomer of conjugated α-linolenic acid, has already demonstrated several health benefits in its chemical characterization, such as acne treatment, reduction of serum lipids, prevention of cancers such as breast and prostate cancer, treatment of type 2 diabetes, and anti-inflammatory properties, among others. Its mechanism of action in the body has not yet been fully elucidated, requiring further studies [146,150].

Pomegranate seeds contain bioactive compounds, such as phenolic compounds, which have been shown to be important for health, acting against various human diseases, such as cancer, diabetes, and cardiovascular diseases. Their phenolic content is 21.01 ± 1.19 µg/mg, as well as 7.46 ± 0.98 of antioxidant activity by the DPPH method. Antioxidants have the capacity to reduce oxidative stress, exhibiting beneficial effects on health [151].

The presence of high levels of tocopherols, such as γ-tocopherol, with an average concentration of 1709.2 mg.kg^−1^, was demonstrated. These act as antioxidants, protecting against oil degradation and acting on the mechanisms of oxidative stress. The main phytosterol found is β-sitosterol, with an average concentration of 1839.69 mg kg^−1^ [150]. Phytosterols are associated with health benefits in relation to hypercholesterolemia, showing a reduction in total cholesterol, triglycerides, and LDL cholesterol in an experimental study with New Zealand mature rabbits [152].

The use of pomegranate seed oil may be beneficial for people (clinical study with 32 men and 48 women with a mean age of 69.53 years) with mild cognitive impairment, improving different domains of cognition, with improvements in visuospatial skills, processing speed and executive functions measured by the Trail Making Test Part B (TMT B), verbal learning and episodic memory measured by the Rey Auditory Verbal Learning Test (RAVLT) and global cognition by the Alzheimer’s Disease Assessment Scale—Cognitive Subscale (ADAS-cog) [153].

Cardiovascular diseases and diabetic complications may present platelet aggregation, advanced glycation end products, and oxidative stress as common key factors for their development. The use of pomegranate and its different parts in an in vitro study demonstrated that it did not show an anti-glycation effect but was able to inhibit platelet aggregation triggered by adenosine diphosphate [75]. In a randomized controlled parallel triple-blind trial, the consumption of symbiotic pomegranate juice was associated with improvements in metabolic and hormonal parameters in women with symptoms of Polycystic Ovarian Syndrome (PCOS), with a mean age of 30 years, as evidenced by a significant reduction in testosterone levels, body mass index (BMI) and body weight [154,155].

In an experimental colitis model, 32 male Sprague-Dawley rats (200–300 g), aged 8–12 weeks, were treated with pomegranate seed oil (PSO) at doses of 0.4 mL/kg and 0.8 mL/kg for 5 days. The results showed that PSO effectively reduced damage to liver and kidney tissues, which are extraintestinal manifestations of colitis, and suppressed NF-κB and apoptosis. PSO treatment at 0.4 mL/kg and 0.8 mL/kg markedly attenuated renal tissue damage [156]. Table 4 summarizes clinical, experimental, and in vitro studies conducted on various products extracted from fruit seeds.

## 5. Limitations and Future Perspectives

The valorization of fruit seed by-products as sources of oils and bioactive compounds represents a rapidly expanding research field; however, several scientific and technological gaps remain to be addressed. Future studies should prioritize the standardization of extraction methodologies, particularly green technologies such as supercritical fluid extraction, ultrasound-assisted extraction, and enzymatic processes, aiming to optimize yield, preserve thermolabile compounds, and ensure reproducibility at an industrial scale. In addition, comparative studies evaluating the efficiency, cost-effectiveness, and environmental impact of these techniques are essential to support their large-scale implementation.

Another relevant perspective involves the comprehensive characterization of bioactive compounds using advanced analytical techniques, such as metabolomics and lipidomic approaches. These strategies can provide more comprehensive information about the chemical complexity of seed-derived matrices and allow the identification of new compounds with potential for nutraceutical, pharmaceutical, cosmetic, or technological applications. From a biological standpoint, although several in vitro and in vivo studies have demonstrated antioxidant, anti-inflammatory, cardioprotective, and metabolic effects, there is still a clear need for well-designed clinical trials to confirm the efficacy, safety, and dose–response relationships of these compounds in humans. The elucidation of molecular mechanisms, particularly involving signaling pathways such as oxidative stress modulation, inflammation, and lipid metabolism, should also be further explored.

In the context of food science and technology, future research should focus on incorporating seed oils and extracts into functional food systems, evaluating their stability, bioavailability, sensory acceptance, and shelf life. Furthermore, encapsulation technologies emerge as a promising strategy to increase the stability and controlled release of bioactive compounds, expanding their applicability in food and pharmaceutical formulations. Furthermore, the integration of fruit seed by-products into circular economy models represents a strategic direction. Studies addressing supply chain optimization, scalability, and techno-economic feasibility are crucial for transforming these residues into high-value-added products. In parallel, beyond the food sector, the development of sustainable applications, such as biofuels, bioplastics, and cosmetic formulations, should be encouraged to maximize the use of these biomasses.

Finally, interdisciplinary approaches that combine food science, biotechnology, environmental science, and industrial engineering will be essential to promote the sustainable exploitation of fruit seed by-products. Above all, establishing alignment between scientific innovation, regulatory frameworks, and market demands will play a decisive role in consolidating these materials as viable alternatives in the global bioeconomy.

## 6. Conclusions

Fruit seeds stand out for their rich chemical composition, which includes vitamins, minerals, bioactive compounds, and fatty acids in significant quantities and of high quality, thereby offering a range of biological and nutritional benefits. As seeds are commonly discarded, managing their use will not only minimize agri-food impacts, such as waste and inadequate disposal of these residues, but will also benefit the food, pharmaceutical, and cosmetics industries, facilitating the development of functional products, as well as biofuels and other biomaterials. Therefore, it is essential to incorporate sustainable practices in the use of fruit seeds, taking into account the principles of the circular economy in the production of natural resources and with a view toward achieving the United Nations Sustainable Development Goals.

## Figures and Tables

**Table 1:** Fatty acid profile of fruit seed oils (g/100 g).

Fatty Acids	Grape Seed Oil [37]	Passion Fruit Seed Oil [38]	Melon Seed Oil [39]	Watermelon Seed Oil [40]	Papaya Seed Oil [41]	Guava Seed Oil [42]	Raspberry Seed Oil [43]	Pomegranate Seed Oil [44]
**Saturated (SFA)**								
Caprylic acid, C8:0	-	-	-	-	-	-	-	-
Lauric acid, C12:0	-	-	-	-	-	-	-	-
Myristic acid, C14:0	0.05	0.1	0.06	-	-	0.56	-	-
Pentadecanoic acid, C15:0	-	-	-	-	-	-	-	-
Palmitic acid, C16:0	8.97	11.72	9.61	16.34	16.95	9.51	3.70	7.73
Heptadecanoic acid, C17:0	-	-	-	-	-	-	-	-
Stearic acid, C18:0	4.04	2.84	-	14.11	3.62	0.16	1.39	2.53
Arachidonic acid, C20:0	-	0.16	-	1.30	-	-	-	3.02
Heneicosanoic acid, C21:0	-	-	-	-	-	0.54	-	1.04
Behenic acid, C22:0	-	0.24	-	-	-	2.72	-	0.77
Lignoceric acid, C24:0	-	-	-	-	-	-	-	-
**∑ Total**	**13.06**	**15.06**	**9.67**	**31.75**	**20.57**	**13.49**	**5.09**	**15.09**
**Monounsaturated (MUFA)**								
Palmitoleic acid, C16:1	0.09	0.34	-	-	0.34	-	-	-
10-heptadecanoic acid, C17:1	-	-	-	-	-	-	-	-
Oleic acid, C18:1n9c	16.75	14.31	15.24	11.44	72.93	7.11	1.36	9.68
Gadoleic acid, C20:1	0.05	0.15	-	-	0.33	-	-	-
Erucic acid, C22:1	-	1.15	-	-	-	-	-	0.92
**∑ Total**	**16.89**	**15.95**	**15.24**	**11.44**	**74.75**	**7.11**	**1.36**	**10.60**
**Poly-Unsaturated (PUFA)**								
Linoleic acid, C18:2n6c	69.0	68.39	73.1	56.81	3.64	75.25	52.37	15.93
Alpha-linolenic acid, C18:3n3	0.44	0.54	-	-	0.31	-	41.18	0.07
Eicosadienoic acid, C20:2	-	-	-	-	0.21	0.39	-	-
Punicic acid, cis-9 trans-11 cis-13 C18:3	-	-	-	-	-	-	-	58.32
**∑ Total**	**69.44**	**68.93**	**73.1**	**56.81**	**4.16**	**75.64**	**93.55**	**74.32**
**∑ SFA + MUFA + PUFA**	**99.39**	**99.94**	**98.01**	**92.67**	**99.48**	**98.24**	**100**	**100**

**Table 2:** Bioactive compounds found in oils extracted from fruit seeds.

Bioactive Compounds	Grape Seed Oil [37]	Passion Fruit Seed Oil [45]	Melon Seed Oil [39]	Watermelon Seed Oil [46]	Papaya Seed Oil [47]	Guava Seed Oil [42]	Raspberry Seed Oil [48]	Pomegranate Seed Oil [44]
Tocopherols (mg/kg)								
α-T	125.1	-	-	-	27.7	-	321.24	25.4
β-T	-	-	-	-	-	-	-	-
γ-T	72.1	5.82	-	-	-	-	550.16	615.6
Δ-T	-	-	-	-	-	-	-	7.8
Tocotrienols (mg/kg)								
α-T3	59.8	-	-	-	-	-	-	-
β-T3	-	-	-	-	-	-	-	-
γ-T3	-		-	-		-	-	-
Δ-T3	-		-	-	-	-	-	-
Phytosterols (mg/100 g)								
Campesterol	-	-	-	-	15.3	-	274.29	-
Stigmasterol	-	-	-	-	9.4	-	147.63	-
β-sitosterol	-	-	-	-	634.4	-	4082.44	-
Δ5-Avenasterol	-	-	-	-	-	-	595.91	-
Δ7-Avenasterol	-	-	-	-	-	-	54.82	-
Total Phenolics (mg/100 g)	-	1.89	29.71	225.64	-	124.03	6.76	14
Total Carotenoids (mg/100 g)	-	-	-	-	-	-	234.41	3.25

**Table 3:** Acidity, peroxide, iodine, saponification and refraction indices of grape, passion fruit, melon, watermelon, papaya, guava, raspberry and pomegranate seed oils.

Indices	Grape Seed Oil [50]	Passion Fruit Seed Oil [36,51]	Melon Seed Oil [52]	Watermelon Seed Oil [40]	Papaya Seed Oil [53]	Guava Seed Oil [42]	Raspberry Seed Oil [54]	Pomegranate Seed Oil [55]
Acidity (mg KOH/g)	-	7.52	0.30	1.63	1.61	0.40	-	-
Peroxide (meq O_2_/kg)	10.75	32.69	0	2.98	1.35	0.62	232	1
Iodine (g I_2_/100 g)	122.05	125.87	-	118.61	64.3	100	-	248.14
Saponification (mg KOH/g)	192.05	184.93	-	187.86	153.96	170	-	167.90
Refractive (40 °C)	1.47	1.47	-	1.472	1.46	1.465	-	-

**Table 4:** Summary of the effects of products extracted from fruit seeds against metabolic dysfunctions.

Reference	Effects	Object/Population	Period	Design	Main Results
[76]	- Cardioprotective - Antioxidant - Anti-inflammatory	Male Wistar-Bratislava rats	14 days	Group 1 (control group): Saline solution 0.4 mL/100 g Group 2 (ISO): Saline solution 0.4 mL/100 g Group 3: Nigella sativa seed oil 0.4 mL/100 g Group 4: Grape seed oil 0.4 mL/100 g	↓ Ventricular conduction ↓ IL-6, IL-1 β and TNF-α ↓ CK-Mb Prevented cardiotoxic effect of ISO
[81]	- Protection against osteoarthritis	Male rats	10 weeks	Group 1: No treatment Group 2: 300 mg/day of piasclidine by mouth Group 3: 1 mg of intra-articular sodium hyaluronate Group 4: 1 mg of intra-articular methylprednisolone acetate Group 5: Avocado oil and grape seed oil (2:1, 300 mg/day) orally Group 6: 500 mg/day of grape seed oil orally Group 7: 200 mg/day of grape seed oil intra-articularly	Prevention of knee osteoarthritis; Effects + medial femoral condyle; +Medial tibial condyle; +Joint space width; +Total number of osteophytes; +On osteoarthritis scores; Results + articular surface; +Cell viability +Calcification
[45]	- Chemopreventive	In vitro (HeLa cells) In vivo (zebrafish xenograft model)	48 h	HeLa cells Group 1: 50 μg/mL of Passiflora edulis f. edulis seed oil (PFSO) Group 2: 100 μg/mL of PFSO Group 3: 200 μg/mL of PFSO Zebrafish embryos Group 1: 4 μg/mL of PFSO Group 2: 8 μg/mL of PFSO Group 3: 16 μg/mL of PFSO Group 4: 32 μg/mL of PFSO	↑ Apoptosis Cell cycle arrest ↓ Antiproliferative effect; ↓ Tumor growth
[98]	- Cardioprotective - Antioxidant - Antidiabetic	Male Wistar rats	15 days	Group 1: Control + saline solution Group 2: Diabetic control + normal saline solution Group 3: Diabetic rats + 0.6 mg/kg of glibenclamide Group 4: Diabetic rats + 250 mg/kg of ethanol extract of passion fruit seeds Group 5: Diabetic rats + 500 mg/kg of ethanol extract of passion fruit seeds	↑ Superoxide dismutase; ↓ 2-thiobarbituric acid-reactive substances ↑ Collagen Inhibition of elastin degradation Inhibition of collagen degradation
[102]	- Cardioprotective - Modulation of the gut microbiota	Hamsters	6 weeks	Group 1: Cholesterol-free diet Group 2: Diet high in cholesterol (0.1%) Group 3: Diet high in cholesterol (4.75% melon seed oil) Group 4: Diet high in cholesterol (9.5% melon seed oil)	↓ Plasma cholesterol Fecal salt excretion ↑ Gene expression of hepatic cholesterol 7α-hydroxylase (CYP7A1) ↑ Short-chain fatty acids Modulation of gut microbiota
[104]	- Cardioprotective - Anti-inflammatory - Antioxidant - Antihypertensive	Mice	28 days	Group 1: Negative control + normal saline (10 mL/kg) Group 2: Control group receiving 5 mg/kg/day of ISO for 14 days, from the 8th to the 21st day of the experiment, along with normal saline administered orally (10 mL/kg). Group 3: Positive control + 10 mg/kg of carvedilol Group 4: Positive control + 10 mg/kg of verapamil Group 5: 75 mg/kg hydroethanolic extract of melon seeds Group 6: 150 mg/kg hydroethanolic extract of melon seeds Group 7: Control with 150 mg/kg hydroethanolic extract of melon seeds	↓ Pulse pressure ↓ Systolic blood pressure ↓ Diastolic blood pressure ↓ Mean arterial pressure ↓ Heart rate ↓ Biometric indicators (cardiac diameter, heart weight, cardiac weight index, left ventricular (LV) weight and thickness, tibia length index, tail length index, and LV index)
[105]	- Cardioprotective - Anti-inflammatory - Antioxidant - Antiatherogenic	Male Wistar rats	12 weeks	Group 1: Control Group 2: High-fat diet (HFD) Group 3: HFD + 50 mg/kg methanolic extract of melon seeds Group 4: HFD + 100 mg/kg methanolic extract of melon seeds Group 5: HFD + 200 mg/kg methanolic extract of melon seeds	↓ Body weight ↓ Body mass index ↓ Brown fat ↓ White fat ↓ Fat mass ↓ Adiposity index ↓ Triglycerides ↓ LDL-C ↓ Total cholesterol ↑ HDL-C
[109]	- Antiteratogenic - Cardioprotective	Male knockout mice	20 weeks	Group 1: Atherogenic diet without supplementation Group 2: Atherogenic diet supplemented with 10% watermelon seed powder	↑ IL-10 ↑ GM-CSF ↑ EPO ↓ MCP-1 ↓ MIP-2 ↓ Average size of atherosclerotic lesions
[110]	- It may have adverse effects on the kidneys and testicles	Male and female Wistar rats	21 days	Group 1: Control Group 2: Diets containing watermelon seeds (WMSs) at 2.5% Group 3: Diets containing watermelon seeds (WMSs) at 5%	↓ Body weight in males ↑ Testicular weight ↓ Ovarian weight ↑ Urea ↑ Creatinine
[118]	- Antioxidant - Immune support - Liver protection	Mice	30 days	Group 1: Control Group 2: Rats fed a basal diet containing 10% chocolate cupcakes with 15% papaya seeds Group 3: Rats received CCl4 + basal diet Group 4: Rats received CCl4 + silymarin (50 mg/kg/day) Group 5: Rats received CCl4 with a basal diet containing 10% chocolate cupcakes with 15% papaya seeds Group 6: Rats received CCl4 and silymarin (50 mg/kg body weight/day) and were fed a basal diet containing 10% cupcakes with 15% papaya seeds	↓ Hepatocellular necrosis Hepatocellular regeneration ↑ Protection against immunosuppression ↑ Protection against hepatotoxicity
[41]	- Cholesterol-lowering agents - Triglyceride-lowering agents - Blood-sugar-lowering agents	Mice	8 weeks	Control Group: 1: Commercial diet + saline solution 2: AIN-93 diet + saline solution 3: High-fat diet + saline solution 4: High-fat diet + soybean oil 5: High-fat diet + olive oil Experimental Group: High-fat diet + papaya seed oil	↓ Total cholesterol ↓ Non-HDL cholesterol ↓ LDL-C ↓ VLDL-C ↑ HDL-C ↓ Fasting blood glucose
[121]	- Neuroprotective - Antioxidant	Albino mice	8 weeks	Group 1: Negative control (normal saline solution) Group 2: Treated with D-galactose (150 mg/kg) Group 3: Positive control (D-galactose (150 mg/kg) and vitamin C (150 mg/kg)) Group 4: D-galactose (150 mg/kg) + papaya pulp extract (PPE) (150 mg/kg) Group 5: D-galactose (150 mg/kg) + papaya seed extract (PSE) (150 mg/kg)	Weight regained ↓ Depression ↓ Anxiety ↑ Muscle strength ↑ GABA in the cortex and hippocampus
[122]	- Safe for oral administration - Antioxidant	Wistar mice	14 days	Oral administration of an ethanol extract of papaya seeds at a dose of 2000 mg/kg body weight	No histopathological abnormalities Normal behavior Stable weight
[135]	- Immunomodulator	In vitro	48 h	PC-3 prostate cancer cells via direct action or indirect tumor immunotherapy using immune-cell-conditioned medium	Inhibition of cell growth ↑ Inhibitory effect on splenocytes ↑ Pro-apoptotic effects Induction of apoptosis
[136]	- Gastric protectant - Antioxidant	Male albino mice	8 days (7 of which were spent acclimatizing)	Group 1: Control (0.5% CMC solution) Group 2: Positive control (0.5% CMC solution) Group 3: Oral cimetidine 100 mg/kg Group 4: Guava seed extract (300 mg/kg)	↓ Ulcerogenic effects Protects mucous membranes Promotes healing ↓ IL-1β ↓ IL-6 ↓ TNF-α
[141]	- Anti-NAFLD (Non-alcoholic fatty liver disease) potential	Male Wistar rats	8 weeks	Group 1: Control (standard diet) Group 2: High-fat diet Group 3: High-fat diet + raspberry seed oil (0.4 mL) Group 4: High-fat diet + raspberry seed oil (0.8 mL)	↓ Insulin resistance ↓ Glucose ↓ Total cholesterol ↓ Triglycerides ↓ LDL-C ↑ HDL-C ↓ ALT ↓ AST ↓ Leptin ↓ NF-κB ↑ PPARγ
[144]	- Cardioprotective - Antioxidant	Young normotensive male Wistar-Kyoto rats and spontaneously hypertensive rats	6 weeks	Group 1: Control group of young normotensive male Wistar-Kyoto rats Group 2: Control group of spontaneously hypertensive rats Group 3: Control group of young normotensive male Wistar-Kyoto rats + 7% ground raspberry seeds Group 4: Control group for spontaneously hypertensive rats + 7% ground raspberry seeds	↓ Aspartate-aspartate aminotransferase ↓ Hydrogen peroxide clearance ↓ Catalase ↓ Atherogenic index ↑ Vasodilation
[153]	- Anti-inflammatory - Antioxidant	Clinical trial Men and women with mild cognitive impairment	1 year of treatment	Control group: Mediterranean diet Experimental group: Mediterranean diet + 5 drops of pomegranate seed oil	Improved performance Improved visuospatial skills Improved processing speed Improved learning Improved memory
[155]	- Antidiabetic - Antioxidant	Women	8 weeks	Control group: Placebo drink Group 1: Symbiotic pomegranate juice Group 2: Pomegranate juice Group 3: Symbiotic drink	↓ Testosterone ↓ BMI ↓ Body weight ↓ Waist circumference Improved insulin resistance
[156]	- Anti-inflammatory - Anti-apoptotic	Male Sprague-Dawley rats	5 days	Group 1: Control Group 2: Colitis + 1 mL of saline solution Group 3: Colitis + treatment with 0.4 mL/kg of pomegranate seed oil Group 4: Colitis + treatment with 0.8 mL/kg of pomegranate seed oil	↓ Liver damage ↓ Kidney damage

↑: increased; ↓: decreased.

## Data Availability

No new data were created or analyzed in this study.
